# Biosurfactant production by *Bacillus subtilis* using corn steep liquor as culture medium

**DOI:** 10.3389/fmicb.2015.00059

**Published:** 2015-02-06

**Authors:** Eduardo J. Gudiña, Elisabete C. Fernandes, Ana I. Rodrigues, José A. Teixeira, Lígia R. Rodrigues

**Affiliations:** CEB – Centre of Biological Engineering, University of MinhoBraga,Portugal

**Keywords:** *Bacillus subtilis*, corn steep liquor, surfactin, bioremediation, microbial enhanced oil recovery

## Abstract

In this work, biosurfactant production by *Bacillus subtilis* #573 was evaluated using corn steep liquor (CSL) as culture medium. The best results were obtained in a culture medium consisting of 10% (v/v) of CSL, with a biosurfactant production of about 1.3 g/l. To the best of our knowledge, this is the first report describing biosurfactant production by *B. subtilis* using CSL as culture medium. Subsequently, the effect of different metals (iron, manganese, and magnesium) on biosurfactant production was evaluated using the medium CSL 10%. It was found that for all the metals tested, the biosurfactant production was increased (up to 4.1, 4.4, and 3.5 g/l for iron, manganese, and magnesium, respectively). When the culture medium was supplemented with the optimum concentration of the three metals simultaneously, the biosurfactant production was increased up to 4.8 g/l. Furthermore, the biosurfactant exhibited a good performance in oil recovery assays when compared with chemical surfactants, which suggests its possible application in microbial enhanced oil recovery or bioremediation.

## INTRODUCTION

Surfactants are an important class of chemical compounds included in many of the everyday products we use. They can be found in detergents, laundry formulations, household cleaning products, cosmetics, herbicides, or pesticides, and are also used in bioremediation, agriculture, food, pharmaceutical, textile, paper, or petroleum industries, among others (Geys et al., 2014; Rebello et al., 2014). Surfactants are amphiphilic molecules that, due to their structure, tend to accumulate at the interfaces between fluid phases with different polarities (e.g., oil-water or air-water), reducing surface and interfacial tensions (Gudiña et al., 2013). Most of the surfactants currently used are chemically synthesized from petrochemical resources and are only partially biodegradable, causing detrimental effects on the environment (Vaz et al., 2012; Rebello et al., 2014).

Nowadays, due to the increasing environmental awareness, the demand for surfactants obtained from bio-based resources is increasing. Biosurfactants, surface-active compounds synthesized by a variety of microorganisms, are attracting a pronounced interest owing to their potential advantages over their chemical counterparts, namely they exhibit a similar or better performance and have less impact on the environment than conventional surfactants, due to their lower toxicity and higher biodegradability. Furthermore, biosurfactants are usually effective at extreme environmental conditions and can be produced from renewable resources (Pereira et al., 2013; Gudiña et al., 2015). As a consequence, biosurfactants can replace some of the synthetic surfactants in many fields. However, the application of biosurfactants depends on whether they can be produced economically at large-scale. Presently, biosurfactants are not competitive with chemical surfactants from an economic point of view, since expensive substrates are required for their production and they present relatively low productivities, which hampers their widespread use and commercialization. As the culture medium can account for up to 30–50% of the overall production costs of biosurfactants, the replacement of expensive synthetic media by cheaper agro-industrial wastes and by-products can contribute to reduce their production costs and increase their competitiveness (Henkel et al., 2012; Al-Bahry et al., 2013; Gudiña et al., 2015).

Biosurfactants display a wide variety of chemical structures, including glycolipids, lipopeptides, phospholipids, fatty acids, or neutral lipids, among others (Gudiña et al., 2013; Geys et al., 2014). *Bacillus* species produce a broad spectrum of lipopeptide biosurfactants, which are cyclic molecules consisting of a fatty acid of variable length (hydrophobic moiety) linked to a short peptide chain (hydrophilic moiety) of seven or ten aminoacids. Among them, surfactin, a lipoheptapeptide produced by *Bacillus subtilis* strains, is one of the most effective biosurfactants known so far; it can reduce the surface tension (ST) of water up to 27 mN/m, with critical micelle concentrations (*cmc*) as low 0.01 g/l, and shows a high emulsifying activity; furthermore, it exhibits antimicrobial, antiviral, and anti-tumor activities (Gudiña et al., 2013). Consequently, surfactin can be potentially useful in numerous therapeutic, industrial, and environmental applications. In order to reduce the production costs, surfactin production by *B. subtilis* strains has been studied using different substrates, such as molasses (Abdel-Mawgoud et al., 2008; Joshi et al., 2008; Al-Bahry et al., 2013), potato peels (Das and Mukherjee, 2007), whey powder (Cagri-Mehmetoglu et al., 2012), cashew apple juice (Oliveira et al., 2013) or cassava waste water (Nitschke and Pastore, 2006). Furthermore, the effect of different nutritional factors has been studied to improve surfactin production by *B. subtilis* strains. Among them, it has been reported that the addition of various metals (i.e., iron, magnesium, and manganese) to the culture medium contribute to increase its production, since those elements are co-factors of enzymes involved in the synthesis of surfactin (Thimon et al., 1992; Makkar and Cameotra, 2002; Wei et al., 2003).

In this work, biosurfactant production by a *B. subtilis* strain was evaluated using the agro-industrial by-product corn steep liquor (CSL) as a low-cost alternative substrate. Once established the optimum CSL concentration for biosurfactant production, the culture medium was optimized by supplementing it with different metals (iron, magnesium, and manganese) at different concentrations, first individually and then in different combinations. Finally, the applicability of the biosurfactant produced in oil recovery was evaluated.

## MATERIALS AND METHODS

### BACTERIAL STRAIN

The biosurfactant-producing strain *B. subtilis* #573 was isolated and identified in our laboratory in a previous work (Gudiña et al., 2012). The strain was stored at –80°C in Luria-Bertani (LB) medium supplemented with 20% (v/v) of glycerol. The composition of LB medium was (g/l): NaCl 10.0; tryptone 10.0; yeast extract 5.0; pH 7.0. Whenever required, frozen stocks were streaked on LB agar plates and incubated at 37°C for 24 h. The agar plates were stored at 4°C no longer than 3 weeks.

### BIOSURFACTANT PRODUCTION BY *B. subtilis* #573 USING CSL

Corn steep liquor was kindly provided by COPAM [Companhia Portuguesa de Amidos, S. A. (Portugal)], and it was evaluated as a substrate for biosurfactant production by *B. subtilis* #573. Total carbohydrates and protein concentrations in CSL were determined using the phenol-sulfuric and Lowry methods, respectively (Lowry et al., 1951; Dubois et al., 1956).

Different culture media were prepared by dissolving CSL in demineralized water at different concentrations [5, 10, and 15% (v/v)]. Subsequently, the pH was adjusted to 7 using 1 M NaOH and the media were sterilized at 121°C for 15 min. Those media were evaluated for biosurfactant production by *B. subtilis* #573 in order to establish the optimum CSL concentration for biosurfactant production. The assays were performed in 500 ml flasks containing 200 ml of each medium. The flasks were inoculated with 2 ml of a pre-culture of *B. subtilis* #573 grown in LB medium at 37°C and 200 rpm for 24 h. Pre-cultures were prepared by inoculating 10 ml of LB medium with a single colony taken from an agar plate. The cultures were incubated at the same conditions (37°C and 200 rpm) until the maximum biosurfactant production was achieved. Samples were taken at different time points during the fermentation to determine biosurfactant production. The samples were centrifuged (4000 rpm, 20 min) and the cell-free supernatants were used to measure the ST and to determine the emulsifying activity (E_24_), as described below. Whenever required, the culture broth supernatants were diluted 10 or 100 times with demineralized water and the surface tension (ST^-1^, ST^-2^) and emulsifying activities (E_24_^-1^, E_24_^-2^) were measured.

### EFFECT OF METAL MEDIUM SUPPLEMENTATION ON BIOSURFACTANT PRODUCTION

Once selected the CSL concentration that led to the highest biosurfactant production by *B. subtilis* #573, the effect of the addition of different metals (iron, manganese, and magnesium) at different concentrations on biosurfactant production was evaluated in order to try to increase biosurfactant production by this isolate. The metal concentrations used were selected according to previous studies performed by different authors, as shown in **Table 1**. Control assays were performed using the culture medium without supplements. The cultures were performed at the same conditions described above and the biosurfactant production was evaluated along the fermentation. The cultures were maintained until the maximum biosurfactant production was achieved in each case.

**Table 1:** Different salts and concentrations used to study the effect of iron, manganese and magnesium on biosurfactant production by *Bacillus subtilis* #573.

Compound	Medium	Concentration	Reference
FeSO_4_	A	0.025 mM	Sen (1997) and Sen and Swaminathan (1997)
	B	0.3 mM	Wei et al. (2007)
	C	2.0 mM	Yeh et al. (2005)

MnSO_4_	D	0.02 mM	Mnif et al. (2012)
	E	0.2 mM	Wei et al. (2007)
	F	2.0 mM	Sen (1997) and Sen and Swaminathan (1997)

MgSO_4_	G	0.8 mM	Sen (1997); Sen and Swaminathan (1997) and Wei et al. (2007)
	H	2.0 mM	Sousa et al. (2012)
	I	4.0 mM	Akpa et al. (2001)

### BIOSURFACTANT RECOVERY

At the end of the fermentation, the cultures were centrifuged (4000 rpm, 20 min) to remove the cells. The cell-free supernatants were adjusted to pH 2 with 6M HCl and were subsequently incubated overnight at 4°C to promote the biosurfactant precipitation. Afterward, the precipitates (crude biosurfactant) were collected by centrifugation (9000 rpm, 20 min, 4°C). The crude biosurfactant was dissolved in a minimal amount of demineralized water and the pH was adjusted to 7 using 1 M NaOH. The biosurfactant solutions were freeze-dried and the products obtained were weighed and stored at –20°C.

### SURFACE-ACTIVITY DETERMINATION

Surface tension measurements of culture broth supernatants and biosurfactant solutions were performed according to the Ring method described elsewhere (Gudiña et al., 2012). A KRÜSS K6 Tensiometer (KRÜSS GmbH, Germany) equipped with a 1.9 cm De Noüy platinum ring was used. To increase the accuracy of the ST measurements, an average of triplicates was determined. All the measurements were performed at room temperature (25°C).

### EMULSIFYING ACTIVITY DETERMINATION

Emulsifying activity was determined by the addition of 2 ml of *n*-hexadecane to the same volume of cell-free supernatants in glass test tubes. The tubes were mixed with a vortex at high speed for 2 min and then incubated at 25°C for 24 h. The emulsification index (E_24_) was calculated as the percentage of the height of the emulsified layer (mm) divided by the total height of the liquid column (mm). All the emulsification indexes were performed in triplicate.

### CRITICAL MICELLE CONCENTRATION (*cmc*)

Critical micelle concentration is the concentration of an amphiphilic compound in solution at which the formation of micelles is initiated. Different concentrations of the freeze-dried biosurfactant produced by *B. subtilis* #573 were prepared in demineralized water, and the ST of each sample was measured at 25°C as described above. The *cmc* was determined by plotting the ST as a function of the logarithm of biosurfactant concentration, and it was found at the point of intersection between the two lines that best fit through the pre- and post-*cmc* data. All the measurements were done in triplicate.

### OIL RECOVERY ASSAYS

The applicability of the biosurfactant produced by *B. subtilis* #573 in oil recovery was evaluated using artificially contaminated sand containing 12.5% (w/w) of Arabian Light crude oil [kindly provided by GALP (Portugal)]. Samples of 40 g of sand were mixed with 5 g of crude oil in 100 ml flasks by shaking, and allowed to age at room temperature for 24 h. Afterward 40 ml of biosurfactant solutions at different concentrations were added to each flask. The flasks were incubated at 90 rpm and 40°C for 24 h, and the oil removed was recovered from the surface and transferred to a graduated tube. In order to separate the water recovered together with the crude oil, the tubes were centrifuged (9000 rpm, 20 min, 25°C), and subsequently the volume of crude oil was measured. The amount of oil recovered (grams) was calculated according to its density (0.837 g/ml). Control assays were performed using demineralized water at the same conditions. All the experiments were performed in triplicate. Statistically significant differences of the assays performed with the different biosurfactant concentrations tested were evaluated by a one-way ANOVA (*P* < 0.05) applying the Tukey multiple-comparisons; a significant difference was considered if *P* < 0.05.

## RESULTS

### EVALUATION OF CSL AS A SUBSTRATE FOR BIOSURFACTANT PRODUCTION

*Bacillus subtilis* #573 was isolated and identified in a previous work as a promising biosurfactant producer (Gudiña et al., 2012). The applicability of the biosurfactant produced by this isolate [identified as a mixture of C_13_-, C_14_-, and C_15_-surfactin (Pereira et al., 2013)] in oil recovery (Pereira et al., 2013) and its anti-tumor activity (Duarte et al., 2014) were also demonstrated. In order to optimize biosurfactant production by this isolate and reduce its production costs, CSL was evaluated as a low-cost alternative culture medium. CSL is a by-product of corn wet-milling, where starch is recovered from corn, and it is an important source of nitrogen for several biotechnological processes (Henkel et al., 2012; Gudiña et al., 2015). The CSL used in this work contained 75 g/l of carbohydrates and 5 g/l of protein.

In order to establish the optimum CSL concentration for biosurfactant production by this isolate, different culture media were prepared by dissolving CSL in demineralized water at different concentrations [5, 10, and 15% (v/v)], and subsequently were evaluated for biosurfactant production. The cultures were incubated at 37°C and 200 rpm, and biosurfactant production was evaluated along the fermentation by measuring the ST and the emulsifying activity of the cell-free supernatants. The cultures were maintained until the maximum biosurfactant production was achieved in each case. The results obtained are shown in **Table 2**.

**Table 2:** Surface tension values [ST, ST^**–1**^ and ST^**–2**^ (mN/m)] and emulsifying indexes [E_**24**_, E_**24**_^**–1**^ **(%)**] obtained in cultures performed with *B. subtilis* #573 grown in media containing CSL at different concentrations [5, 10, and 15% (v/v)].

	CSL 5%	CSL 10%	CSL 15%
Time (h)	24	48	48
ST_0_ (mN/m)	51.4 ± 0.4	52.8 ± 0.3	50.5 ± 0.4
ST (mN/m)	32.8 ± 0.5	30.7 ± 0.4	31.8 ± 0.5
ST^-1^ (mN/m)	50.3 ± 1.7	34.9 ± 0.9	40.0 ± 0.8
ST^-2^ (mN/m)	72.0 ± 0.0	59.8 ± 1.8	67.3 ± 1.5
E_24_ (%)	28.0 ± 3.7	55.0 ± 2.0	40.3 ± 1.7
E_24_^-1^ (%)	0.0	29.0 ± 6.6	0.0

*The results presented correspond to the values obtained at the optimum time for each medium. ST_0_: surface tension of the culture medium. Results represent the average of three independent experiments ± SD.*

Although with the three media similar ST values were obtained in the cell-free supernatants without dilution, more pronounced differences were observed in the cell-free supernatants 10 and 100 times diluted. With the medium CSL 5%, the best results regarding biosurfactant production were obtained after 24 h, whereas for the other two media these were obtained after 48 h. From **Table 2** it can be seen that the lowest ST values and the highest emulsifying indexes were obtained with the medium CSL 10%, thus indicating a higher biosurfactant production. Taking into account the price of CSL (about 40€/ton), the price of 1 liter of medium CSL 10% is about 0.004€. Therefore, the use of CSL as an alternative culture medium would greatly decrease the production costs of this biosurfactant at a higher production scale.

The cell-free supernatants of cultures performed in CSL 10% for 48 h were subjected to acidic precipitation to recover the biosurfactant produced, yielding 1.311 ± 0.109 g of biosurfactant per liter. The *cmc* calculated for this biosurfactant was 0.16 g/l. In view of the results obtained, the medium CSL 10% was selected to perform the following optimization assays.

### EFFECT OF METALS ON THE BIOSURFACTANT PRODUCTION

Iron, manganese, and magnesium are co-factors of enzymes involved in the synthesis of surfactin by *B. subtilis*. Therefore, the concentration of these elements in the culture medium plays an important role in the biosurfactant production (Sen, 1997; Wei and Chu, 1998, 2002; Wei et al., 2003, 2007). The medium CSL 10%, selected in the previous phase as the best medium for biosurfactant production by *B. subtilis* #573, was supplemented with FeSO_4_, MnSO_4,_ or MgSO_4_ individually at different concentrations (as shown in **Table 1**), in order to study their effect on biosurfactant production. The metal concentrations used were selected taking into account previous works from other authors studying biosurfactant production by different *B. subtilis* strains. The medium CSL 10% without supplements was used as control. As in the previous assays, the cultures were incubated at 37°C and 200 rpm, and biosurfactant production was evaluated along the time. The cultures were maintained until the maximum biosurfactant production was achieved in each case. The results obtained are gathered in **Table 3**.

**Table 3:** Surface tension values [ST, ST^**–1**^, and ST^**–2**^ (mN/m)], emulsifying indexes [E_**24**_, E_**24**_^**–1**^ (%)] and biosurfactant concentrations obtained in cultures performed with *B. subtilis* #573 grown in CSL 10% supplemented with different metals at different concentrations.

Medium*(time)	ST (mN/m)	ST^1^(mN/m)	ST^2^(mN/m)	E_24_(%)	E_24_^1^(%)	[BS] (g/l)
A (72 h)	31.0 ± 0.7	33.7 ± 1.4	55.8 ± 3.0	54.5 ± 1.0	31.5 ± 2.5	1.915 ± 0.124
B (48 h)	30.6 ± 0.5	33.3 ± 1.0	55.1 ± 1.7	56.5 ± 0.6	25.3 ± 1.0	1.991 ± 0.100
C (72 h)	29.7 ± 0.5	32.6 ± 0.6	46.8 ± 1.0	57.0 ± 1.2	41.0 ± 2.0	4.170 ± 0.054
D (72 h)	30.4 ± 0.5	32.5 ± 1.0	48.2 ± 1.0	57.0 ± 3.5	32.0 ± 2.8	3.690 ± 0.135
E (72 h)	30.4 ± 0.5	33.8 ± 1.0	46.0 ± 0.9	59.0 ± 2.0	41.0 ± 2.6	4.467 ± 0.158
F (72 h)	30.7 ± 1.0	32.3 ± 0.5	47.6 ± 1.3	57.8 ± 0.5	31.5 ± 1.9	3.901 ± 0.061
G (72 h)	30.8 ± 0.9	34.1 ± 3.3	48.7 ± 1.2	58.0 ± 2.3	43.0 ± 6.0	3.519 ± 0.102
H (48 h)	30.0 ± 0.1	32.6 ± 1.5	49.0 ± 0.5	55.0 ± 1.2	28.0 ± 2.8	3.405 ± 0.167
I (48 h)	30.3 ± 0.5	32.5 ± 0.4	53.1 ± 1.7	56.3 ± 2.1	21.5 ± 1.9	2.381 ± 0.170
Control (48 h)	30.7 ± 0.4	34.9 ± 0.9	59.8 ± 1.8	55.0 ± 2.0	29.0 ± 6.6	1.311 ± 0.109

Control: CSL 10% without supplements.ST_0_: 52.8 ± 0.3 mN/m. ST_0_^-1^: 60.3 ± 0.5 mN/m. ST_0_^-2^: 70.0 ± 0.8 mN/m. The results presented correspond to the optimum time for each medium. Results represent the average ± SD of three independent experiments. *The composition of the different culture media is shown in **Table 1**.

For the experiments conducted with the media B, H, and I, the lowest ST values were obtained at 48 h of growth, as it was previously observed in the assays performed with the medium CSL 10%. In the other media, the lowest ST values were achieved at 72 h of growth. Regarding the ST of the cell-free supernatants without dilution, similar values (between 29.7 and 31.0 mN/m) were obtained for all the media tested. However, greater differences were observed in the cell-free supernatants that were diluted 10 and 100 times (**Table 3**).

In the culture media supplemented with FeSO_4_, the best result was obtained with the highest concentration tested (2.0 mM, medium C). In that case, the ST^-2^ was reduced up to 46.8 ± 1.0 mN/m at 72 h, which resulted in a biosurfactant production of 4.170 ± 0.054 g/l. Regarding manganese, the best result was obtained with MnSO_4_ at a concentration 0.2 mM (medium E) at 72 h. The ST^-2^ was reduced up to 46.0 ± 0.9 mN/m, and the amount of biosurfactant produced was 4.467 ± 0.158 g/l. In the media supplemented with magnesium, similar results were obtained with MgSO_4_ 0.8 and 2.0 mM (media G and H). The ST^-2^ was reduced up to 48.7–49.0 mN/m, and the amount of biosurfactant produced ranged between 3.4 and 3.5 g/l. In all the cases a direct relationship was found between the ST^-2^ values and the amount of biosurfactant produced (**Table 3**).

Regarding the emulsifying indexes, similar values (54.5–59.0%) were obtained with all the media assayed (**Table 3**). Emulsifying indexes higher than 50% entail that the hydrocarbon phase is completely emulsified. More pronounced differences were observed for the E_24_^-1^ values. The highest E_24_^-1^ values were obtained with the media C, E, and G, which is in agreement with the amounts of biosurfactant produced in each medium.

As it can be seen from the results obtained, the supplementation of the medium CSL 10% with the different metals resulted in an increase in biosurfactant production in all the cases when compared with the medium without supplements, although for each metal, that effect was dependent on the concentration used.

It has been reported that the combination of different metals can result in a positive interaction on surfactin production as compared with their individual effect (Sen, 1997; Makkar and Cameotra, 2002). Taking into account the results obtained with the medium CSL 10% supplemented with the different metals individually, this medium was supplemented with different metal combinations, according with the concentrations that led to the best results in the individual assays for each compound, namely FeSO_4_ 2.0 mM, MnSO_4_ 0.2 mM, and MgSO_4_ 0.8 mM. The different combinations assayed are summarized in **Table 4**. As in the previous assays, the cultures were incubated at 37°C and 200 rpm, and biosurfactant production was evaluated along the time. The medium CSL 10% without supplements was used as control. The cultures were maintained until the maximum biosurfactant production was achieved in each case. The results obtained are gathered in **Table 5**.

**Table 4:** Combinations of metal salts assayed for studying its effect on the biosurfactant production by *B. subtilis* #573.

Medium	Compound	Concentration
J	FeSO_4_	2.0 mM
	MgSO_4_	0.8 mM

K	FeSO_4_	2.0 mM
	MnSO_4_	0.2 mM

L	MgSO_4_	0.8 mM
	MnSO_4_	0.2 mM

M	FeSO_4_	2.0 mM
	MgSO_4_	0.8 mM
	MnSO_4_	0.2 mM

**Table 5:** Surface tension valuesmN/m)], emulsifying indexes [E_**24**_, E_**24**_^**–1**^ (%)] and biosurfactant concentrations obtained in cultures performed with *B. subtilis* #573 grown in CSL 10% supplemented with different combinations of metals.

Medium*(time)	ST (mN/m)	ST^-1^(mN/m)	ST^-2^(mN/m)	E_24_(%)	E_24_^-1^ (%)	[BS] (g/l)
J (24 h)	29.3 ± 0.4	31.6 ± 0.5	51.6 ± 2.0	55.5 ± 1.9	34.0 ± 3.7	2.704 ± 0.132
K (24 h)	29.3 ± 0.3	31.5 ± 0.8	47.4 ± 1.9	56.5 ± 1.0	34.0 ± 2.3	3.933 ± 0.205
L (48 h)	29.7 ± 0.3	31.3 ± 1.0	46.6 ± 1.3	57.0 ± 1.2	26.0 ± 1.6	4.224 ± 0.157
M (72 h)	29.1 ± 0.6	31.3 ± 0.9	45.1 ± 1.9	59.5 ± 0.9	36.1 ± 1.7	4.829 ± 0.193
Control (48 h)	30.7 ± 0.4	34.9 ± 0.9	59.8 ± 1.8	55.0 ± 2.0	29.0 ± 6.6	1.311 ± 0.109

Control: CSL 10% without supplements. ST_0_: 52.8 ± 0.3 mN/m. ST_0_^-1^: 60.3 ± 0.5 mN/m. ST_0_^-2^: 70.0 ± 0.8 mN/m. The results presented correspond to the optimum time for each medium.Results represent the average ± SD of three independent experiments. *The composition of the different culture media is shown in **Table 4**. [ST, ST^–1^, and ST^–2^

Regarding the combinations iron-magnesium (medium J) and iron-manganese (medium K), the best results regarding ST reduction and biosurfactant production were obtained at 24 h of growth. However, the amount of biosurfactant produced (2.704 ± 0.132 and 3.933 ± 0.205 g/l, respectively) was lower than the obtained when those metals were added individually, which suggests a negative interaction on the biosurfactant production. Regarding the combination magnesium-manganese (medium L), the best results were obtained after 48 h. In this case, the amount of biosurfactant produced (4.224 ± 0.157 g/l) was slightly lower when compared with the obtained with the optimum concentration of manganese, but better than the obtained with the optimum concentration of magnesium. Finally, a combination of the three metals was tested (medium M). The best results were obtained at 72 h, and the amount of biosurfactant produced (4.829 ± 0.193 g/l) was higher than the obtained when the different metals were added individually.

## OIL RECOVERY ASSAYS

The oil recovery assays were performed using the freeze-dried biosurfactant produced by *B. subtilis* #573 in the medium CSL 10%, dissolved in demineralized water at different concentrations. The results obtained are shown in **Table 6**.

**Table 6:** Percentages of oil recovered with the biosurfactant produced by *B. subtilis* #573 in the medium CSL 10% at different concentrations.

[BS] g/l	Oil recovered (%)
5.0	25.1 ± 1.7
2.5	26.3 ± 0.0
1.0	15.0 ± 0.8
Control	0.0 ± 0.0

*Results represent the average ± SD of three independent experiments.*

As it can be seen from **Table 6**, similar oil recoveries were obtained with the biosurfactant at a concentration of 2.5 and 5.0 g/l [the differences were not statistically significant (*P* > 0.05)], which suggests that the use of higher biosurfactant concentrations would not increase the oil recovery.

## DISCUSSION

Biosurfactant production by *B. subtilis* isolates using low-cost substrates was studied by several authors. *B. subtilis* B20 and B30 produced between 0.3 and 2.3 g/l of biosurfactant using a mineral medium supplemented with date molasses as carbon source (Al-Bahry et al., 2013; Al-Wahaibi et al., 2014). Abdel-Mawgoud et al. (2008) reported a biosurfactant production of 1.12 g/l by *B. subtilis* BS5 using a mineral medium containing sugarcane molasses. *B. subtilis* LAMI005 produced 0.3 and 0.44 g/l of biosurfactant growing in media containing cashew apple juice (Oliveira et al., 2013) and raw glycerol derived from biodiesel production (Sousa et al., 2012), respectively. However, in these cases, other nutrients (e.g., carbon or nitrogen sources, salts) were added to the media, thus increasing their prices. On the contrary, in the present work, the only substrate used to prepare the culture media was CSL. Likewise, other authors reported biosurfactant production by different *B. subtilis* isolates using culture media containing exclusively agro-industrial by-products (e.g., cassava wastewater, sugarcane molasses, or rehydrated whey powder), with productivities between 0.3 and 3.0 g/l (Nitschke and Pastore, 2006; Joshi et al., 2008; Cagri-Mehmetoglu et al., 2012). However, to the best of our knowledge, this is the first report describing biosurfactant production by *B. subtilis* using CSL as culture medium. The amount of biosurfactant produced (about 1.3 g/l) was close to the values reported by other authors using different substrates.

It has been reported that the addition of iron, magnesium or manganese to the culture medium increased biosurfactant production by *B. subtilis* isolates (Thimon et al., 1992; Wei and Chu, 1998, 2002; Makkar and Cameotra, 2002; Al-Ajlani et al., 2007). Mn^2+^ at a concentration 0.01 mM increased biosurfactant production by *B. subtilis* ATCC 21332 from 0.33 to 2.6 g/l (Wei and Chu, 2002). Al-Ajlani et al. (2007) reported an increase in biosurfactant production by *B. subtilis* MZ-7 from 0.22 to 0.3 g/l by supplementing the culture medium with Fe^2+^ at a concentration 4.0 mM; and similar results were obtained by Thimon et al. (1992). Makkar and Cameotra (2002) increased biosurfactant production by *B. subtilis* MTCC 2423 growing in a mineral medium from 0.342 to 0.814 g/l by the addition of FeSO_4_ (0.719 mM), and to 0.792 g/l by the addition of MgSO_4_ (2.43 mM). However, in some cases, higher concentrations of those metals inhibited biosurfactant production (Makkar and Cameotra, 2002). In our case, the amount of biosurfactant produced by *B. subtilis* #573 in the medium CSL 10% was increased up to 3.2, 3.4, and 2.7 times due to the addition of iron, manganese, and magnesium, respectively, to the culture medium.

Sen (1997) reported a strong and positive interaction between iron and manganese in the biosynthesis of surfactin by *B. subtilis*. Wei et al. (2007) increased biosurfactant production by *B. subtilis* ATCC 21332 from 1.74 to 3.34 g/l through the optimization of the trace elements composition (Mg^2+^, K^+^, Mn^2+^, Fe^2+^, and Ca^2+^). Makkar and Cameotra (2002) also reported an increase on biosurfactant production by *B. subtilis* MTCC 2423 from 0.342 to 1.230 g/l when the culture medium was supplemented with the optimum concentrations of magnesium, iron, and calcium simultaneously, and this effect was higher than the obtained when the different metals were added individually. In our case, a combination of FeSO_4_ 2.0 mM, MgSO_4_ 0.8 mM, and MnSO_4_ 0.2 mM increased biosurfactant production 3.6 times as compared with the medium without supplements.

Regarding the oil recovery assays, in a previous work, the biosurfactant produced by this isolate in a mineral medium (containing sucrose as the carbon source and ammonium nitrate as the nitrogen source) recovered 18.8 ± 1.1% of oil at a concentration of 1 g/l (Pereira et al., 2013). That value is higher than the obtained in the current work (15.0 ± 0.8%) at the same conditions. However, the *cmc* calculated for the biosurfactant produced in the mineral medium by this isolate was 0.03 g/l (Pereira et al., 2013), whereas for the biosurfactant produced in CSL 10% it was 0.16 g/l. This can explain the differences observed in the percentages of oil recovered in both cases. Nevertheless, the results obtained in this work are still better comparing with the oil recoveries obtained with the chemical surfactants Enordet and Petrostep at the same concentrations and conditions (9–12%; Pereira et al., 2013), thus suggesting that this biosurfactant can be useful for application in microbial enhanced oil recovery or bioremediation.

## CONCLUSION

Biosurfactant production by *B. subtilis* #573 was evaluated using CSL as an alternative low-cost culture medium. The use of such substrate led to a surfactin production of about 1.3 g/l, which is in good agreement with the values reported so far in the literature. To our knowledge this is the first report on the use of CSL for the production of surfactin. Besides, if an industrial scale is envisaged, the use of CSL as culture medium would have a huge impact in the surfactin production costs. Additionally, the amount of biosurfactants produced using this alternative medium was increased about 3 to 4 times when metal salts were used as supplements, thus representing an even higher impact on the production costs. It is important to mention that the surfactin produced from these alternative media retained all its previously reported properties, including its great potential for microbial enhanced oil recovery or bioremediation applications.

## AUTHOR CONTRIBUTIONS

All authors contributed to the design of the work. Eduardo J. Gudiña, Elisabete C. Fernandes and Ana I. Rodrigues performed the experimental work and wrote part of the manuscript. José A. Teixeira and Lígia R. Rodrigues participated in the design of the study and drafted the manuscript. All authors read and approved the final manuscript.

## Conflict of Interest Statement

The authors declare that the research was conducted in the absence of any commercial or financial relationships that could be construed as a potential conflict of interest.
